# A Hybrid Newton–Raphson and Particle Swarm Optimization Method for Target Motion Analysis by Batch Processing

**DOI:** 10.3390/s21062033

**Published:** 2021-03-13

**Authors:** Raegeun Oh, Yifang Shi, Jee Woong Choi

**Affiliations:** 1 Department of Marine Science & Convergence Engineering, Hanyang University ERICA, Ansan 15588, Korea; rgoh@hanyang.ac.kr; 2 School of Automation, Hangzhou Dianzi University, Xiasha Higher Education Zone, 2nd Street, Hangzhou 310018, China; syf2008@hdu.edu.cn

**Keywords:** bearing-only target motion analysis, batch estimation, hybrid optimization

## Abstract

Bearing-only target motion analysis (BO-TMA) by batch processing remains a challenge due to the lack of information on underwater target maneuvering and the nonlinearity of sensor measurements. Traditional batch estimation for BO-TMA is mainly performed based on deterministic algorithms, and studies performed with heuristic algorithms have recently been reported. However, since the two algorithms have their own advantages and disadvantages, interest in a hybrid method that complements the disadvantages and combines the advantages of the two algorithms is increasing. In this study, we proposed Newton–Raphson particle swarm optimization (NRPSO): a hybrid method that combines the Newton–Raphson method and the particle swarm optimization method, which are representative methods that utilize deterministic and heuristic algorithms, respectively. The BO-TMA performance obtained using the proposed NRPSO was tested by varying the measurement noise and number of measurements for three targets with different maneuvers. The results showed that the advantages of both methods were well combined, which improved the performance.

## 1. Introduction

Sonar systems used to detect and track underwater targets can be divided into active sonar or passive sonar, depending on the sensor operating mode. Active sonar transmits sound waves and receives reflected or scattered signals from the target to find the target direction and measure the echo time in order to infer the range of the target. Passive sonar receives the signal generated by the target, but can only measure the target direction excluding the echo time [1]. As a result of the lack of information on target maneuvering and the nonlinearity of sensor measurements, the design of a reliable algorithm for bearing-only target motion analysis (BO-TMA) remains a challenge.

The bearing of the target measured from passive sonar is the basic information used for performing target motion analysis (TMA), and TMA performed with the maneuvering information of the observer and the target bearing information gathered from passive sonar is divided into two types: batch estimation and sequential estimation. Sequential estimation can analyze the target state for real-time usage by utilizing the target bearing acquired at each time. However, for good estimation convergence, batch estimation that can estimate an accurate target initial state value is necessary [2]. For sequential estimation for BO-TMA, it is common to extend the Bayesian filter to a nonlinear system by using an extended Kalman filter (EKF) [3,4]. In addition, various sequential estimation algorithms utilizing a particle filter (PF) [5] and the interacting multiple model (IMM) [6] have been proposed to obtain effective sub-optimal solutions for sequential BO-TMA. For batch estimation, a maximum likelihood estimator (MLE) utilizing the Newton–Raphson method (NRM) is widely used for BO-TMA. This utilizes a batch of accumulated bearing information to form a quadratic cost function that can be minimized by utilizing the weighted squares of errors [7,8,9]. The MLE generates a solution using an iterative method; therefore, it is computationally more demanding. Subsequently, a pseudo-linear estimator (PLE) based on reconstructing the original nonlinear bearing measurement into a linear measurement form of the unknown target state has been proposed [10,11]. This estimator does not rely on iterative processing and it is not time consuming; however, it is known to produce biased estimates. The performance of both sequential and batch estimations can be sensitive to their initialization. Traditional batch estimation for BO-TMA is mainly based on a deterministic algorithm, but such methods have often failed to find the target state due to an inaccurate initialization method. Recently, studies on heuristic algorithms that are relatively free from initialization problems have been introduced. Genetic algorithm (GA), a heuristic algorithm, was used in the batch estimation to solve the BO-TMA problem [12]. The performance of the modified EKF (MEKF) was improved compared to that of the traditional EKF when using heuristic algorithms (i.e., particle swarm optimization, genetic algorithm, cuckoo search) for batch estimation [13]. Oh et al. [2] used the particle swarm optimization (PSO) method to estimate the optimal initial target state vector from the bearing lines produced by correcting the bearing errors caused by the multipath of the underwater target. In general, heuristic algorithms can be performed for a large range of target state variations, regardless of the initial target state covariance matrix, and they can be applied to problems where differentiation is not possible. 

Currently, hybrid methods combining different types of methods are being studied in various fields. In order to accurately predict the relative humidity in the atmosphere in a specific geographical location, a hybrid model combining the physics-based numerical weather prediction (NWP) model and the data-driven Gaussian process (GP) model has been proposed [14]. In addition, a study was conducted to find a combination of usual industrial radioactive sources that achieves the highest precision in dual-energy radiation-based three-phase flow meters, in which a hybrid model, an adaptive neural fuzzy reasoning system (ANFIS) trained through gray wolf optimization (GWO), was used [15]. In the field of electrical impedance tomography, the quality of the reconstructed image was improved by optimizing the radial basis function neural network (RBFNN) with the hybrid particle swarm optimization (HPSO) algorithm [16]. A multimodel deep learning (MMDL) framework has been proposed that takes into account the strengths of a deep autoencoder neural network (DeepAEC) and one-dimensional conventional neural network (1D-CNN) to effectively enhance the performance of the recommender system (RS) [17]. However, BO-TMA that uses a hybrid method combining deterministic and heuristic methods has not yet been reported. 

The goal of this paper is to obtain a reliable initial target position and velocity using the proposed batch estimation algorithm from the noise-corrupted bearing measurements. For this purpose, we proposed a hybrid method that combines a deterministic method and a heuristic method. In this paper, Section 2 defines several BO-TMA scenarios to check the performance of batch estimation. In Section 3, NRM and PSO are briefly introduced; these are representative methods that utilize deterministic and heuristic algorithms. A hybrid method that combines these two algorithms is also introduced. The BO-TMA performances of these three algorithms are compared under various conditions in Section 4. Finally, a summary and conclusion are given in Section 5.

## 2. Problem Formulation

BO-TMA is a method to estimate the target state, including the target position and speed, using a batch of bearing measurements obtained from a passive sonar system (e.g., a horizontal line array sonar system). It is possible to estimate the trajectory of the target by assuming rectilinear and uniform motion of the target in Cartesian coordinates from a batch of measurements contaminated by random noise. The mathematical model for calculating the target bearing requires the positions of the target and the sensor at each discrete measuring time. The target position can be calculated by the following state equation:

(1) $$X_{s}\left(k+1\right)=FX_{s}\left(k\right)+GU_{s}\left(k\right),$$

where the target state vector at the discrete time instance k, $X_{s}\left(k\right)$, consists of a two-dimensional position and velocity components ${\left[p_{xs}\left(k\right),p_{ys}\left(k\right),v_{xs}\left(k\right),v_{ys}\left(k\right)\right]}^{T}$, and the control input, $U_{s}\left(k\right)$, is a two-dimensional target acceleration ${\left[u_{xs}\left(k\right),\;u_{ys}\left(k\right)\right]}^{T}$. The subscript s indicates the target. The observer state can also be described by the same form as in Equation (1) with the subscript o instead of the subscript s. F and G are the state transition matrix and the input coefficient matrix, respectively [2], as follows:

(2) F=[I2ΔtI202I2], G=[Δt22I2ΔtI2],

where $I_{2}$ is the two-dimensional identity matrix, $0_{2}$ is the 2×2 zero matrix, and Δt is the time interval. In general, $U_{s}\left(k\right)$ for every time instance k, 1≤k≤K, is zero due to the assumption of rectilinear and uniform motion of the target, which represents a constant velocity (CV) model. The $U_{o}\left(k\right)$ of the observer has a specific value to ensure system observability [3].

The true target bearing at time k can be defined by the following nonlinear equation:

(3) $$\theta \left(k\right)=h\left(X_{s}\left(k\right)\right)=atan2\left(p_{xs}\left(k\right)-p_{xo}\left(k\right),\;p_{ys}\left(k\right)-p_{yo}\left(k\right)\right),$$

where atan2(x, y) denotes a four-quadrant arctangent function that describes the angle from the observer to the target measured in the clockwise positive direction from the north axis (y-axis). The bearing of the target is typically modeled as being corrupted by the random measurement noise γ(k), which is modeled by independent zero mean Gaussian noise with standard deviation σ. The bearing measurement is modeled by the following equation:

(4) z(k)=θ(k)+γ(k).

If there is no measurement noise, the measurement becomes the target true bearing, and the initial state vector of the target can be estimated through the accumulated measurements along the time instance with the own-ship maneuver to enhance system observability. However, if the random measurement noise is considered, the bearing error due to the measurement noise is reflected as an estimation error of the target initial state vector. Therefore, the BO-TMA with the measurement noise is considered a problem of estimating the initial state vector of the target with the minimum error. For the random measurement noise simulated with a Gaussian distribution, in general, the larger the standard deviation, the greater the estimation error. Moreover, since the mean value of the measurement noise is zero, the greater the number of measurements, the smaller the estimation error.

In this paper, it was assumed that the right/left ambiguity that may arise in a horizontal line array sonar system was resolved through an observer maneuver. In addition, the underwater target signal could be received by a multipath with multiple elevation angles; however, for simplicity, a direct path with a horizontal bearing angle only was considered for measurements [2]. The target bearing obtained as a result of signal processing was used as target information in the BO-TMA, and it was assumed that the targets were all detected in the simulated time instance. The performance of BO-TMA may vary depending on the signal processing method, signal to noise ratio, and the positional error of the observer; however, these problems are beyond the scope of this study, and thus only the performance of BO-TMA due to measurement noise was considered in this paper.

**Figure 1:**
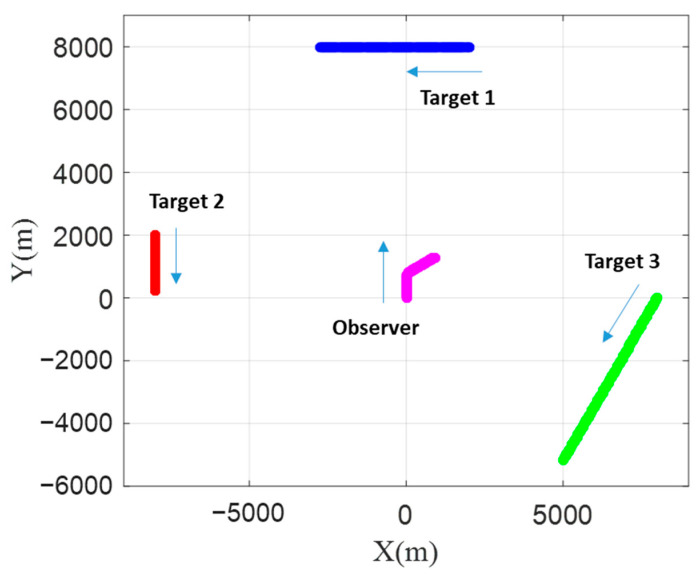
Trajectories of Target 1, Target 2, Target 3, and the observer in the horizontal plane.

BO-TMA is performed with variations of the bearing measurements over time, and the variations of the bearing depend on the relative position between the observer and the target. Therefore, in this paper, in order to verify the performance of BO-TMA in various situations, three targets with different histories of bearings, headings, and speeds were designed. Assuming that each target did not appear at the same time, the BO-TMA problems of the three scenarios are independent. The horizontal plane trajectories of the targets and the observer are shown in Figure 1, and the detailed information is shown in Table 1. The total simulation time was 600 s with various sampling periods for the different number of measurements. The initial state vector of the observer, $X_{o}\left(1\right),$ was [0 km, 0 km, 0 m/s, 3 m/s]. To ensure system observability, the course of the observer was changed once from 0° to 60° via lateral acceleration starting at 200 s. The bearing change rate was 0.6°/s. The initial state vectors of Target 1, Target 2, and Target 3 with zero acceleration over the simulation time were [2 km, 8 km, −8 m/s, 0 m/s], [−8 km, 2 km, 0 m/s, −3 m/s], and [8 km, 0 km, −5 m/s, −8.7 m/s], respectively. 

## 3. Methodology

### 3.1. Cost Function

For BO-TMA by batch estimation, the maximum likelihood estimator (MLE) can be used to estimate target state variables given the history of measured bearings $Z={\left[z\left(1\right),\;\cdots ,z\left(K\right)\right]}^{T}$. Since the random measurement noise is zero mean Gaussian noise, the least squares cost function to be minimized through an iterative manner is formed with the measured bearings Z, the bearings of each time instance calculated from the assumed target initial state, and the known observer states. For the BO-TMA problem in this paper, the quadratic cost function is defined as [7]

(5) $$J\left(\hat{X}\right)=\sum_{k=1}^{K}\left[{\left\{z\left(k\right)-h\left(\hat{X}\left(k\right)\right)\right\}}^{T}W^{-1}\left\{z\left(k\right)-h\left(\hat{X}\left(k\right)\right)\right\}\right],$$

where $\hat{X}$ is an estimated state that is expected to be the initial target state at k=1 and W is the covariance of the bearing measurement noise. In general, in batch processing techniques, the difference between the measurements and the estimated bearings is normalized with the standard deviation of measurement error, and the square sum of this value is used as the cost function [7,9,10,12].

Optimization methods are divided into deterministic and heuristic methods. In this paper, the performances of NRM (a deterministic method) and PSO (a heuristic method) were analyzed to find a robust solution that provides the minimum cost function for a given scenario. A hybrid method that combined the advantages of the two algorithms was also proposed.

### 3.2. Newton–Raphson Method

Deterministic methods like NRM aim to implement an iterative algorithm that utilizes gradients of the cost function to induce the minimum of the cost function after a certain number of iterations. The iterative algorithm used in this method is presented below [18,19]:

(6) $${\hat{X}}^{l+1}={\hat{X}}^{l}-\left[{\left(\frac{\partial M}{\partial {\hat{X}}^{l}}\right)}^{-1}M\right],$$

(7) $$M=\frac{\partial J\left({\hat{X}}^{l}\right)}{\partial {\hat{X}}^{l}}=\sum_{k=1}^{K}\left[{\left\{-\frac{\partial h\left({\hat{X}}^{l}\left(k\right)\right)}{\partial {\hat{X}}^{l}}\right\}}^{T}W^{-1}\left\{z\left(k\right)-h\left({\hat{X}}^{l}\left(k\right)\right)\right\}\right],$$

where ${\hat{X}}^{l}$ is the l-th iterative estimated initial target state at k=1. While the steepest descent and conjugate gradient methods use the first derivative of the cost function for iteration, NRM uses the second derivative (known as the Hessian matrix) to achieve faster convergence. However, it requires the inverse of the Hessian matrix, which makes the method even more computationally expensive, and it may diverge for cases with large initialization errors, an insufficient number of measurements, or poor geometry between the observer and the targets. 

For effective NRM implementation, appropriate initialization of the iterative algorithm is required. Therefore, to find the appropriate initial state of the target at l=1, it is necessary to restrict the possible range of the state value, as shown in Table 2. In this paper, to find the accurate initial state of the iterative search, the best state that produced the minimum cost value was selected among 10,000 random candidates within a limited search area.

### 3.3. Particle Swarm Optimization

Particle swarm optimization (PSO) is a metaheuristic optimization algorithm that was proposed by Kennedy and Eberhart [20]. PSO aims to imitate the social interactions of animals moving in flocks, rather than individual cognitive abilities. This approach has been used in a variety of fields because it is easily applicable, even to complex multidimensional problems that cannot be solved using traditional deterministic algorithms [21,22,23]. For BO-TMA by batch estimation, the application of a deterministic method is common, although batch estimation with a heuristic method has been recently proposed. The process is expressed as [2]

(8) $${\hat{X}}_{i}^{l+1}={\hat{X}}_{i}^{l}+v_{i}^{l+1},$$

(9) $$v_{i}^{l+1}=\alpha v_{i}^{l}+c_{l}R\left(Lbest_{i}^{l}-{\hat{X}}_{i}^{l}\right)+c_{g}R\left(Gbest^{l}-{\hat{X}}_{i}^{l}\right),$$

where ${\hat{X}}_{i}^{l}$ and $v_{i}^{l}$ represent the estimated initial target state and the state change rate of the i-th particle for the l-th iteration at k=1, respectively. The total number of particles and the total number of iterations are 400 and 50, respectively. The local state vector $Lbest_{i}^{l}$ is the best state vector of the i-th particle obtained from the first iteration to the l-th iteration, and the social state vector $Gbest^{l}$ is the best state vector of the particle with the smallest cost value J of all particles up to the l-th iteration. R indicates a 4 × 4 diagonal matrix with random numbers between 0 and 1 as its elements. The acceleration constants α, $c_{l}$, and $c_{g}$ are the inertial weight, cognitive coefficient, and social coefficient, respectively. 

The performance of the PSO algorithm is heavily influenced by the three acceleration constants α, $c_{l}$, and $c_{g}$. The basic role of α is to determine the global and local search capabilities. A large α facilitates a global search, while a small α facilitates a local search. $c_{l}$ and $c_{g}$ represent the extent to which particles are directed to the individual and global best states, respectively. A small acceleration constant restricts the movement of particles, while a large acceleration constant can cause the divergence of the swarm. Therefore, it is necessary to appropriately determine each acceleration constant according to the characteristics of the problem to be solved in the search space.

**Figure 2:**
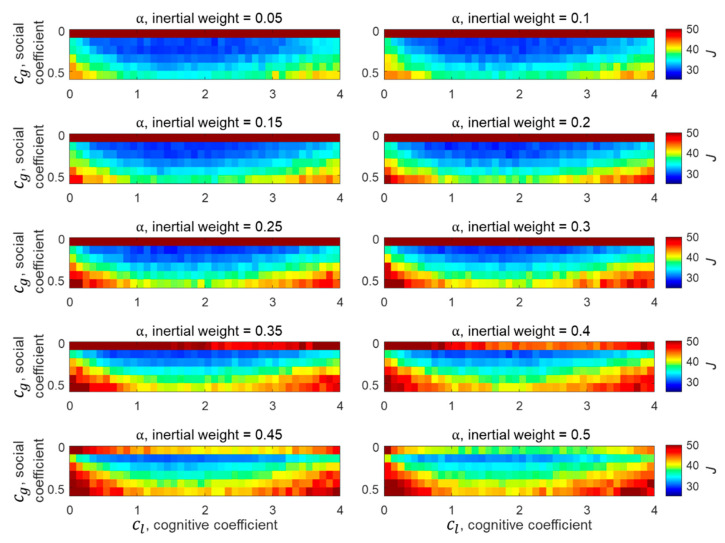
Simulation results for determining the acceleration constants α, $c_{l}$, and $c_{g}$ in the final search spaces.

Since PSO was initially proposed, many studies have been conducted to determine the values of the three acceleration constants [20,21,22]. However, since no investigation has been conducted on the optimal acceleration constants for BO-TMA, an analysis was performed here to determine the optimal acceleration constant values. For this, BO-TMA using the PSO method for Target 1 was performed with random measurement noise corresponding to σ=0.1° for each discrete set of acceleration constants within two search spaces of α, $c_{l}$, and $c_{g}$. The initial search spaces between 0.05 and 2.00 for α and between 0.05 and 4.05 for $c_{l}$ and $c_{g}$ were first examined at an interval of 0.05 for α and intervals of 0.2 for $c_{l}$ and $c_{g}$. On the basis of the results of this first stage of analysis, narrow final search spaces for the three acceleration constants were established. These were 0.05≤α≤0.50, $0.05\leq c_{l}\leq 4.05$, and $0.05\leq c_{g}\leq 0.60$ at intervals of 0.05, 0.1, and 0.1, respectively. For each set of acceleration constants, 500 random noises were generated within a range of the measurement noise of σ=0.1°, and the cost function for 500 random noises was calculated and averaged to obtain a mean cost function. Finally, the acceleration constant set that produced the minimum mean cost function was selected. Figure 2 shows the simulation results with various acceleration constant values. The values that produced the minimum cost function were α=0.25, $c_{l}=1.55$, and $c_{g}=0.15$. The small α and $c_{g}$ values and large $c_{l}$ values in the simulation results indicate that even if particles converge slowly within the search range, they tend to allow for a precise search by properly weighting each particle’s individual experience. However, three acceleration constants may be analyzed differently as the other PSO parameters, i.e., total number of particles and number of iterations, are changed. As the total number of particles increases, more particles contribute to search within the search range, resulting in more precise analysis. Therefore, depending on the total number of particles, the weights of global and local searches may be changed.

### 3.4. Newton–Raphson Particle Swarm Optimization

NRM, a deterministic method considered in the BO-TMA problem, has the advantage of being able to derive accurate estimates in a short time by reacting sensitively to the given batch of measurements. However, because it is too sensitive to the quality of measurements, the error in the estimate can increase significantly as the measurement noise increases. In addition, even if the initialization is set within the possible range of the state vector of the target, the estimated state vector may exceed the possible range due to divergence. Since NRM is not a global search algorithm, there is the problem that it may converge to a local solution, depending on the initialization. PSO is considered to be a heuristic method that has the ability to search the entire area within the allowable range of variations; it can also be utilized for BO-TMA. This method generates a solution with convergence based on the experience of each particle. Even if the measurement noise is large, the target state vector can be estimated within the allowable range. However, it is not guaranteed to produce optimal results.

The hybrid optimization algorithm proposed in this paper, i.e., Newton–Raphson particle swarm optimization (NRPSO), combines the advantages of NRM and PSO in BO-TMA. Interest in hybrid optimization methods has increased over the past few decades [14,15,16,17,22,23,24]. Early hybridization was mainly done between several metaheuristic algorithms. In recent years, research on systems of cooperation between metaheuristic and deterministic methods has increased. Hybrid optimization algorithms typically use heuristic methods to generate good candidates for the optimal solution, and then automatically switch to deterministic methods to converge to the optimal solution. However, deterministic methods performed on noisy measurements still have the potential to diverge. Therefore, NRPSO is conducted in a parallel manner for NRM and PSO algorithms. The process of performing the NRPSO method is similar to the conventional PSO method, but the difference is that the weighting factor by the NRM method is added to the state change rate when determining the particles of the next generation in the PSO method. The process of NRPSO, in which Equation (6) for NRM and Equation (9) for PSO are synthesized, is expressed as follows:

(10) $${\hat{X}}_{i}^{l+1}={\hat{X}}_{i}^{l}+nrpso\_v_{i}^{l+1},$$

(11) $$nrpso\_v_{i}^{l+1}=v_{i}^{l+1}+c_{n}R\left(NRM_{i}^{l+1}\right),$$

(12) $$NRM_{i}^{l+1}=\left[{\left(\frac{\partial M}{\partial {\hat{X}}_{i}^{l}}\right)}^{-1}M\right],$$

where $NRM_{i}^{l+1}$ denotes the calculated amount of change of the state vector using NRM at the i-th particle ${\hat{X}}_{i}^{l}$ for the l-th iteration, and M is defined in Equation (7). The acceleration constant $c_{n}$ is an NRM coefficient and was set to 0.1, which is smaller than the social coefficient to ensure convergence of particles. As can be seen from the equation, NRPSO works by adding the direction of descent, which is based on the local curvature of the cost function, to the search for the individual cognitive and social coefficients at each iteration. 

The proposed NRPSO solves the problem based on the experience of all particles as the number of iterations increases, similar to PSO. At the same time, each particle utilizes the local curvature of the cost function for convergence, as shown in the case of NRM. However, NRPSO reveals divergence in estimating the target state sometimes, unlike PSO. This divergence is due to the role of NRM in NRPSO, which involves the calculation of the second derivative of the cost function. Therefore, NRPSO requires an adjustment between NRM and PSO by utilizing the determinant of the Hessian matrix. In the NRM method, when the value of this determinant is close to zero, it is difficult to find an optimal solution, and the case is designated to be “ill-conditioned” [25]. That is, for the “ill-conditioned” case where the result of NRM may be unstable, only PSO is performed. Alternatively, for the “well-conditioned” case, PSO and NRM are performed together, and stable results are expected. Therefore, Equation (11) of the NRPSO algorithm is changed as follows to coordinate the two methods.

(13) $$nrpso\_v_{i}^{l+1}=\{\begin{array}{cc} v_{i}^{l+1}+c_{n}R\left(NRM_{i}^{l+1}\right), & if\;\left|\frac{\partial M}{\partial {\hat{X}}_{i}^{l}}\right|\geq N_{thr}\\ v_{i}^{l+1}, & if\;\left|\frac{\partial M}{\partial {\hat{X}}_{i}^{l}}\right|<N_{thr} \end{array},$$

where $N_{thr}$ represents the constant threshold for determining the “ill-conditioned” case. The result of NRPSO becomes similar to that of PSO as $N_{thr}$ increases, and becomes similar to that of NRM as $N_{thr}$ decreases. The value of $N_{thr}$ can be determined according to the problem to be solved. For the BO-TMA problem in this paper, the value of $10^{-4}$ was assigned empirically through many trial runs. In order to compare the estimation performance of the hybrid model with the models performed individually, the other parameters of NRPSO were set to be equal to those of NRM and PSO. The BO-TMA result obtained by NRPSO with the selected $N_{thr}$ value is presented in Section 4, and it can be confirmed that the advantages of the NRM method and the PSO method are well combined.

## 4. Simulation Results

In this section, we compare the performance of NRM, PSO, and the proposed NRPSO for the aforementioned scenario. The bearing measurements in Section 2 and the three batch estimation algorithms for performing BO-TMA were simulated using the MATLAB code. The initial states of the three targets were selected within the allowable range of the values, as shown in Table 2. The BO-TMA results relative to the measurement noise and the number of measurements were derived for each target. The standard deviation of the measurement noise considered here was selected from 0.1° to 1.9° with increments of 0.2°, and the number of measurements was selected from 100 to 600 with increments of 100. Finally, all results for the three targets were assessed as BO-TMA performance over 1000 Monte Carlo simulation runs. Equations for evaluating the BO-TMA performance are defined as follows:

(14) $$Position\;RMSE=\sqrt{\frac{\sum \left\{{\left(p_{xs}-{\hat{p}}_{xs}\right)}^{2}+{\left(p_{ys}-{\hat{p}}_{ys}\right)}^{2}\right\}}{N}},$$

(15) $$Velocity\;RMSE=\sqrt{\frac{\sum \left\{{\left(v_{xs}-{\hat{v}}_{xs}\right)}^{2}+{\left(v_{ys}-{\hat{v}}_{ys}\right)}^{2}\right\}}{N}},$$

(16) $$Percent\;of\;divergence=\frac{number\;of\;divergence}{Total\;number\;of\;Monte\;Carlo\;simulation\;runs}\times 100,$$

where $\left[{\hat{p}}_{xs},{\hat{p}}_{ys},{\hat{v}}_{xs},{\hat{v}}_{ys}\right]$ is the estimated initial state vector of BO-TMA, and N is the number of Monte Carlo simulation runs that did not diverge. The BO-TMA performance using the three methods was evaluated by the root mean square error (RMSE) values of the position and velocity between the true target state and the estimated state. Divergence occurred when the estimated position was more than 20 km away from the observer. 

**Figure 3:**
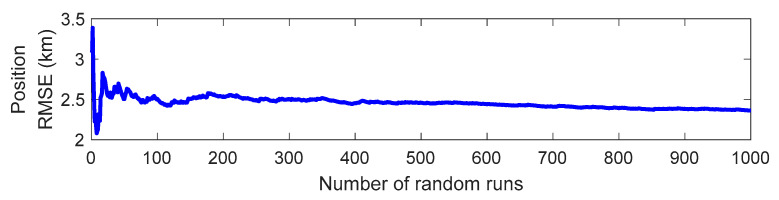
Convergence of the position root mean square error (RMSE) of Target 2 obtained by Newton–Raphson particle swarm optimization (NRPSO), in which 600 bearing measurements corrupted by measurement noise with a standard deviation of 1.9° were used.

In BO-TMA, even if the target bearings of the scenario are the same, the error of the initial state vector between the estimated target and the true target may be small or large as a result of the included random measurement noise. Evaluating the performance of the BO-TMA from a single run result is not suitable because the error of the initial state vector fluctuates significantly as the standard deviation of the measurement noise increases. Therefore, in this paper, a Monte Carlo simulation was conducted to ensure statistically reliable BO-TMA performance from measurements including random noise [2,3,4,7,9,10,11,13]. Figure 3 shows the convergence of the position RMSE of Target 2 obtained by NRPSO using 600 bearing measurements corrupted by the measurement noise with a standard deviation of 1.9°. As shown in Figure 3, cases including fluctuations in the RMSE with a small number of runs (i.e., <100 runs) were avoided; thus, a Monte Carlo simulation with 1000 runs was performed for each BO-TMA. The number of simulation runs was determined by examining the variation of the position RMSE with respect to the number of runs. 

**Figure 4:**
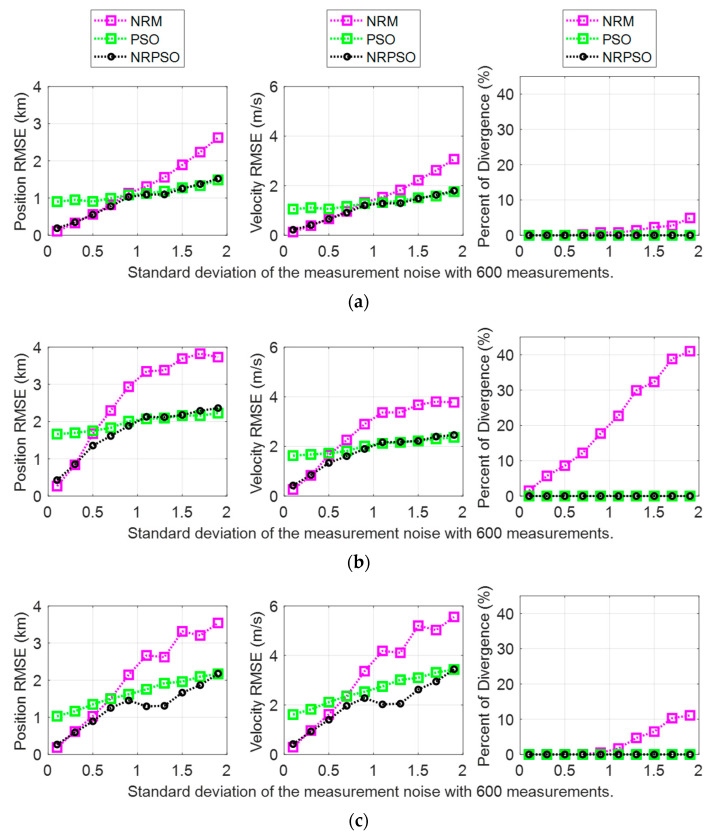
Bearing-only target motion analysis (BO-TMA) results versus measurement noise standard deviation for (**a**) Target 1, (**b**) Target 2, and (**c**) Target 3. The results of the Newton–Raphson method (NRM), particle swarm optimization (PSO), and NRPSO are represented by magenta, green, and black, respectively. The left column indicates the position RMSE, the center column shows the velocity RMSE, and the right column shows the rate of divergence.

The BO-TMA results showed similar trends for the three targets with various standard deviations of measurement noise with 600 measurements. These results are shown in Figure 4. The NRM method showed accurate estimates with small standard deviations without divergence, but it generated the “ill-conditioned” Hessian matrix as the standard deviation increased, indicating that the position RMSE of the estimate increased. In the case of Target 2, the rate of divergence rapidly increased by up to about 40% when the standard deviation of the measurement noise was 1.9°. Here, divergence refers to a case where the target state cannot be found due to inaccurate initialization. In this paper, divergence was classified as a case where the estimated distance to the target from the observer was more than 20 km. The PSO method showed a larger RMSE than the NRM method for cases where the measurement noise had a small standard deviation, but the increase in the RMSE in accordance with the increase of the standard deviation remained small. Consequently, for cases where the measurement noise had a large standard deviation, PSO generated the target state estimates with a smaller RMSE than the NRM. In particular, PSO did not show divergence for all standard deviations, which is important for checking the reliability of the estimation results. These results are caused by the fact that NRM applies the local curvature of the cost function and generates the solution outside the allowable range of state variations specified in Table 1 as the number of iterations increases, whereas PSO only considers the solution inside the search area. The results of the NRPSO method showed a good combination of the advantages of the deterministic and heuristic methods. It was confirmed that NRPSO tended to generate the smaller RMSE of the two methods for all standard deviations, and it did not show divergence. 

**Figure 5:**
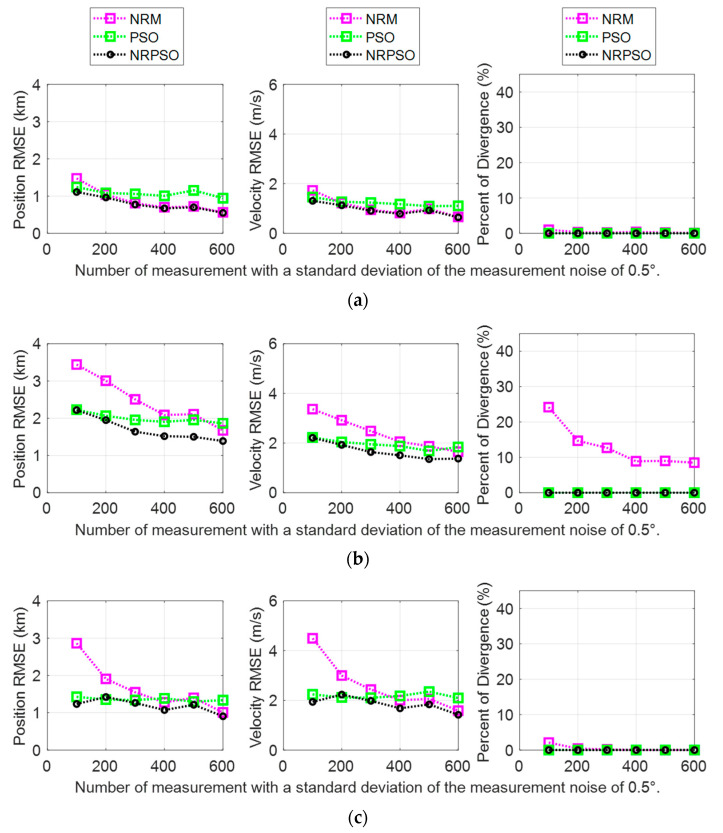
BO-TMA results versus number of measurements when the standard deviation of the measurement noise was 0.5° for (**a**) Target 1, (**b**) Target 2, and (**c**) Target 3. The results of NRM, PSO, and NRPSO are represented by magenta, green, and black, respectively. The left column shows the position RMSE, the center column shows the velocity RMSE, and the right column shows the rate of divergence.

The BO-TMA performance was also confirmed using various numbers of measurements according to the sampling period with a measurement noise of σ=0.5°. These results are shown in Figure 5. The measurement noise was assumed to be a zero mean Gaussian noise. Therefore, the case with fewer measurements is closer to the “ill-conditioned” case, and the case with a larger number of measurements is closer to the “well-conditioned” case. It was confirmed that NRPSO selected the result with the smaller error between the two methods for all of the cases with different numbers of measurements, and it did not show divergence. As a result, it was confirmed that NRPSO worked robustly for various conditions.

**Figure 6:**
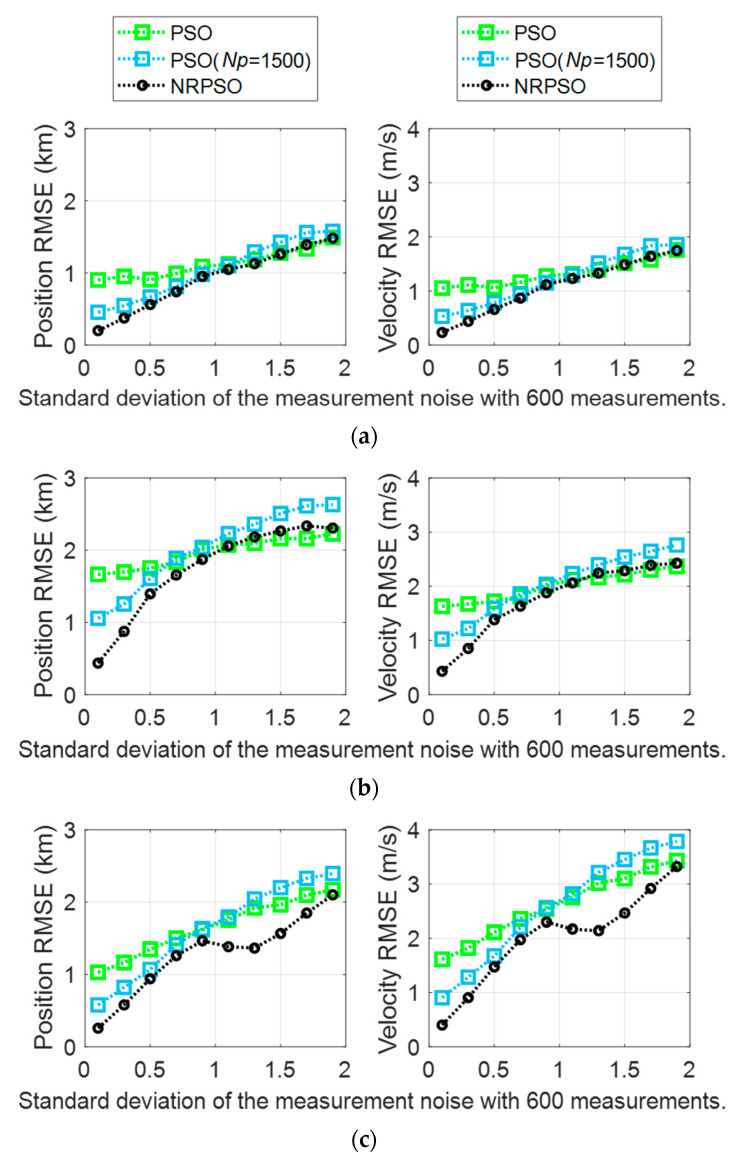
BO-TMA results versus measurement noise standard deviation for (**a**) Target 1, (**b**) Target 2, and (**c**) Target 3. The results of PSO (Np=400), PSO (Np=1500), and NRPSO (Np=400) are represented by green, blue, and black, respectively. The left column indicates the position RMSE, and the right column shows the velocity RMSE.

In this paper, a hybrid method was proposed to derive a reliable initial target position and velocity from noise-corrupted bearing measurements. In the previously presented results, the BO-TMA results of NRPSO showed better estimation performance than the case where NRM and PSO were utilized individually. In general, the amount of computational power and the performance of the optimization method are proportional. NRPSO requires a large amount of computational power because it is performed by combining the NRM method and the PSO method. Therefore, additional simulations were performed to compare the performance of NRPSO and PSO at a similar computational power. As the number of particles in the PSO method increases, more computational power is required, and more precise estimation can be obtained. To increase the amount of computational power of PSO, the number of particles, Np, was increased from 400 to 1500 so that the CPU time for each run in the PSO process was similar to that of the NRPSO. The results are listed in Figure 6. The results of PSO with Np=1500 showed smaller RMSEs for smaller measurement noise standard deviations than the results of PSO with Np=400; however, it showed increased RMSEs for larger standard deviations. Therefore, the estimation performance of PSO became similar to NRM as the number of particles increases. For all the standard deviations in consideration, the RMSEs of NRPSO were smaller than those of PSO with Np=1500. In conclusion, even with a similar amount of computational power, NRPSO still shows better estimation performance than PSO.

## 5. Summary and Conclusions

In this paper, the BO-TMA results of NRM and PSO were compared for various conditions. For the BO-TMA problem, appropriate values of input parameters of the PSO method were determined through a series of simulation studies. The convergence rate of the PSO method was shown to be slow, although more robust estimation results were obtained. 

The NRM method performed accurately when the standard deviation of the measurement noise was small or when the number of the measurements was large. However, in the opposite cases, the heuristic method (i.e., PSO) produced more accurate estimation results than NRM. Moreover, divergence, which may be caused by inaccurate initialization of NRM, did not occur with PSO. After analyzing the BO-TMA results of NRM and PSO, they were transformed into a hybrid form called NRPSO that combined the advantages of the two methods. Through a series of simulation studies, it was found that NRPSO can be used to estimate the initial target states under various conditions with accuracy and robustness, without the divergence that often occurs in NRM. 

It should be noted that the computational load was not considered in this paper. However, NRPSO requires significant computation power in order to run many iterations with a sufficiently large number of particles to perform accurate target localization. Therefore, sufficient computation power is required for practical scenarios that demand fast computation. If this is not possible, an appropriate compromise can be found between the computation burden and accuracy, or NRPSO can be applied to only part of the overall iteration process. In order to effectively utilize the NRPSO method proposed in this paper, a study on the degradation of the estimation performance due to the reduction in the computational load is required, and a comparison with various batch estimation methods is needed to verify the NRPSO performance.

## Figures and Tables

**Table 1:** Observer and target information.

Parameter	Observer	Target 1	Target 2	Target 3
Initial position ($p_{x},p_{y}$)	0 km, 0 km	2 km, 8 km	−8 km, 2 km	8 km, 0 km
Initial speed	3 m/s	8 m/s	3 m/s	10 m/s
Initial heading (second heading)	0° (60°)	270°	180°	210°
Target range variation	-	7.2–8.2 km	8.0–9.0 km	7.2–8.0 km
Target bearing variation	-	−29–14°	−97–−76°	90–148°

**Table 2:** Search range of the state variables.

Parameter	Value
Euclidean distance	0–15 km
Speed	0–15 m/s
Heading	−180–180°
Bearing	z(1)±3σ
